# Timing, Indicators, and Approaches to Digital Patient Experience Evaluation: Umbrella Systematic Review

**DOI:** 10.2196/46308

**Published:** 2024-02-05

**Authors:** Tingting Wang, Guido Giunti, Richard Goossens, Marijke Melles

**Affiliations:** 1 Department of Human-Centered Design Faculty of Industrial Design Engineering Delft University of Technology Delft Netherlands; 2 Research Unit of Health Sciences and Technology Faculty of Medicine University of Oulu Oulu Finland; 3 Clinical Medicine Neurology School of Medicine Trinity College Dublin Dublin Ireland

**Keywords:** digital health, eHealth, telemedicine, mobile health, mHealth, patient experience, user experience, evaluation timing, evaluation indicators, evaluation approaches, user-centered design, patient-centered care, human-computer interaction, mobile phone

## Abstract

**Background:**

The increasing prevalence of DH applications has outpaced research and practice in digital health (DH) evaluations. Patient experience (PEx) was reported as one of the challenges facing the health system by the World Health Organization. To generate evidence on DH and promote the appropriate integration and use of technologies, a standard evaluation of PEx in DH is required.

**Objective:**

This study aims to systematically identify evaluation timing considerations (ie, *when to measure*), evaluation indicators (ie, *what to measure*), and evaluation approaches (ie, *how to measure*) with regard to digital PEx. The overall aim of this study is to generate an evaluation guide for further improving digital PEx evaluation.

**Methods:**

This is a 2-phase study parallel to our previous study. In phase 1, literature reviews related to PEx in DH were systematically searched from Scopus, PubMed, and Web of Science databases. Two independent raters conducted 2 rounds of paper screening, including title and abstract screening and full-text screening, and assessed the interrater reliability for 20% (round 1: 23/115 and round 2: 12/58) random samples using the Fleiss-Cohen coefficient (round 1: *k*1=0.88 and round 2: *k*2=0.80). When reaching interrater reliability (*k*>0.60), TW conducted the rest of the screening process, leaving any uncertainties for group discussions. Overall, 38% (45/119) of the articles were considered eligible for further thematic analysis. In phase 2, to check if there were any meaningful novel insights that would change our conclusions, we performed an updated literature search in which we collected 294 newly published reviews, of which 102 (34.7%) were identified as eligible articles. We considered them to have no important changes to our original results on the research objectives. Therefore, they were not integrated into the synthesis of this review and were used as supplementary materials.

**Results:**

Our review highlights 5 typical evaluation objectives that serve 5 stakeholder groups separately. We identified a set of key evaluation timing considerations and classified them into 3 categories: intervention maturity stages, timing of the evaluation, and timing of data collection. Information on evaluation indicators of digital PEx was identified and summarized into 3 categories (intervention outputs, patient outcomes, and health care system impact), 9 themes, and 22 subthemes. A set of evaluation theories, common study designs, data collection methods and instruments, and data analysis approaches was captured, which can be used or adapted to evaluate digital PEx.

**Conclusions:**

Our findings enabled us to generate an evaluation guide to help DH intervention researchers, designers, developers, and program evaluators evaluate digital PEx. Finally, we propose 6 directions for encouraging further digital PEx evaluation research and practice to address the challenge of poor PEx.

## Introduction

### Background

Emerging digital technologies promise to shape the future health care industry [1,2]. According to our previous review [3], most researchers had a positive impression of digital health interventions (DHIs). The number of DHIs is proliferating [4-6], which is affecting the way patients receive their health care services compared with face-to-face health care services and ultimately influencing the patient journey and overall patient experience (PEx) [7,8]. Good PEx is a key intent of patient-centered care [9] and a core measure of care quality in digital health (DH) [10,11]. Digital technologies have the potential to enhance or provide comparable PEx compared with some face-to-face health care services [8,12-14]. However, the uptake of digital technologies in health care is not as rapid as it has been in many other industries [15], and their potential in health care remains unfulfilled [16]. According to a report by the World Health Organization (WHO) on the classification of DHIs, the health system is not responding adequately to the need for improved PEx [17].

Despite the growing number of DHIs, evaluations that are timely, cost-effective, and robust have not kept pace with this growth [7,18,19]. PExs in the wide range of DHIs are mixed [20,21]. Few published DHIs have resulted in high download numbers and active users [22]; most are released with minimal or no evaluation and require patients to assess the quality for themselves and take responsibility for any consequences [23]. Low-quality DH may disrupt user experience (UX) [24], resulting in low acceptance, and some may even be harmful [25]. In addition, a DHI may be popular with patients but not valued by clinicians [26]. To generate evidence and promote the appropriate integration and use of digital technologies in health care, an overview of how to evaluate PEx or UX in varied DHIs is needed [3,27].

### Evaluating the Digital PEx

In this study, we used the definition of digital PEx from our previous review [3]: “the sum of all interactions affected by a patient’s behavioral determinants, framed by digital technologies, and shaped by organizational culture, that influence patient perceptions across the continuum of care channeling digital health.” This incorporates influencing factors of digital PEx [3] and the existing definitions of DHIs [28,29], PEx [30], and UX [31]. Compared with the general PEx and UX, it highlights patient perceptions that are affected by technical, behavioral, and organizational determinants when interacting with a DHI. DHI has become an umbrella term that often encompasses broad concepts and technologies [32], such as DH applications, ecosystems, and platforms [28]. In this study, we followed the WHO’s definition of DHIs [29], that is, the use of digital, mobile, and wireless technologies to support the achievement of health objectives. It refers to the use of information and communication technologies for health care, encompassing both mobile health and eHealth [29,33]. Compared with evaluating DHIs, PEx, and UX, little is known about evaluating digital PEx. However, combining the definition of digital PEx with the extensively explored measurement of PEx, UX, and DHIs can lead to an improved understanding of and enable the development of evaluation approaches for measuring digital PEx. Therefore, the evaluations of PEx, UX, and DHIs will be used as a starting point in this study to clarify when to measure, what to measure, and how to measure digital PEx.

### When to Measure

First, the timing of measuring and evaluating digital PEx is an important consideration and must align with the contextual situation, such as evaluation objectives and stakeholders, to ensure practicality and purposefulness [34,35]. According to the European Union [36] and the Department of Health of The King’s Fund [37], an evaluation can be scheduled during the design phase or during or after the implementation phase. Similarly, the WHO [29] introduced 3 DHI evaluation stages: efficacy, effectiveness, and implementation. The evaluation of efficacy refers to where the intervention is under highly controlled conditions, the evaluation of effectiveness is carried out in a real world context, and the evaluation of implementation occurs after efficacy and effectiveness have been established. Furthermore, an evaluation can be performed before, during, or after the evaluated intervention in both research and nonresearch settings [36]. However, decision-making on *when* to collect PEx data can be more complicated. As argued in earlier studies [35,37], immediate feedback has the benefit of gaining real-time insights, but patients may be too unwell, stressed, or distracted to provide detailed opinions. In contrast, when the feedback is related to medical outcomes or quality of life, it often requires a lengthy period after the intervention to observe any changes. However, responses gathered long after a care episode may be inferior because of recall bias.

### What to Measure

Second, there is a need for a decision on *what* is required to measure to assess digital PEx. The frequently mentioned UX evaluation concepts, such as usability, functionality, and reliability, from studies [38-40] investigating UX can be applied to evaluate the intervention outputs to anticipate digital PEx at a service level. Moreover, according to the existing constructs and frameworks of understanding or evaluating PEx [41-45], such as emotional support, relieving fear and anxiety, patients as active participants in care, and continuity of care and relationships, they can be adjusted to evaluate digital PEx by understanding patient outcomes at an individual level. In addition, the National Quality Forum [11] proposed a set of measurable concepts to be used to evaluate PEx in telehealth, for example, patients’ increased confidence in, understanding of, and compliance with their care plan; reduction in diagnostic errors and avoidance of adverse outcomes; and decrease in waiting times and eliminated travel. Some of these concepts can be used to understand digital PEx at an organizational level by assessing the impact of the health care system.

### How to Measure

The third consideration is *how* to choose evaluation approaches appropriate for evaluating the digital PEx [35], starting from widely used theories, study designs, methods, and tools for evaluating DHIs and the related PEx or UX. There is rapidly evolving guidance for guiding DH innovators [18], such as the National Institute for Health and Care Excellence Evidence Standards Framework for Digital Health Technologies [46]. The strength of the evidence in the evaluation of DHIs often depends on the study design [18]. However, the high bar for evidence in health care usually requires a longer time for evidence generation, such as prospective randomized controlled trials (RCTs) and observational studies, which often conflicts with the fast-innovation reality of the technology industry [16,18]. In addition, many traditional approaches, such as qualitative and quantitative methods, can be used to collect experience-related data to evaluate the DHIs [18,29]. Qualitative methods such as focus groups, interviews, and observations are often used to obtain an in-depth understanding of PEx [37] in the early intervention development stages [29]. Surveys using structured questionnaires, such as patient satisfaction ratings [37,47], patient-reported experience measures (PREMs) [35,48], and patient-reported outcome measures (PROMs) [35,37,48], are often used to examine patterns and trends from a large sample. Hodgson [49] believed that strong evidence results from UX data that are valid and reliable, such as formative and summative usability tests, and stated that behavioral data are strong, but opinion data are weak.

### Objectives

This study aims to systematically identify (1) evaluation timing considerations (ie, *when to measure*), (2) evaluation indicators (ie, *what to measure*), and (3) evaluation approaches (ie, *how to measure*) with regard to digital PEx. The overall aim of this study is to generate an evaluation guide for further improving digital PEx evaluation research and practice.

## Methods

### Overview

This study consists of 2 phases. In phase 1, we followed the same study search and selection process as our previous research [3] but focused on a different data extraction and analysis process to achieve our objectives in this study. In the previous study [3], we identified the influencing factors and design considerations of digital PEx, provided a definition, constructed a design and evaluation framework, and generated 9 design guidelines to help DH designers and developers improve digital PEx. To highlight the connections between “design” and “evaluation” works in the development of DH and provide readers with a clear road map, we included some evaluation-related information in the previous paper as well. However, it was limited and described at a very abstract level. In this study, detailed information on the evaluation was provided, including evaluation timing considerations, evaluation indicators, and evaluation approaches, and we aimed to generate an evaluation guide for improving the measurement of digital PEx. Given that this is an evolving area, after we finished phase 1, we conducted an updated literature search as a subsequent investigation to determine whether an update of a review was needed in this study.

### Phase 1: The Original Review

#### Study Search and Selection

**Figure 1:**
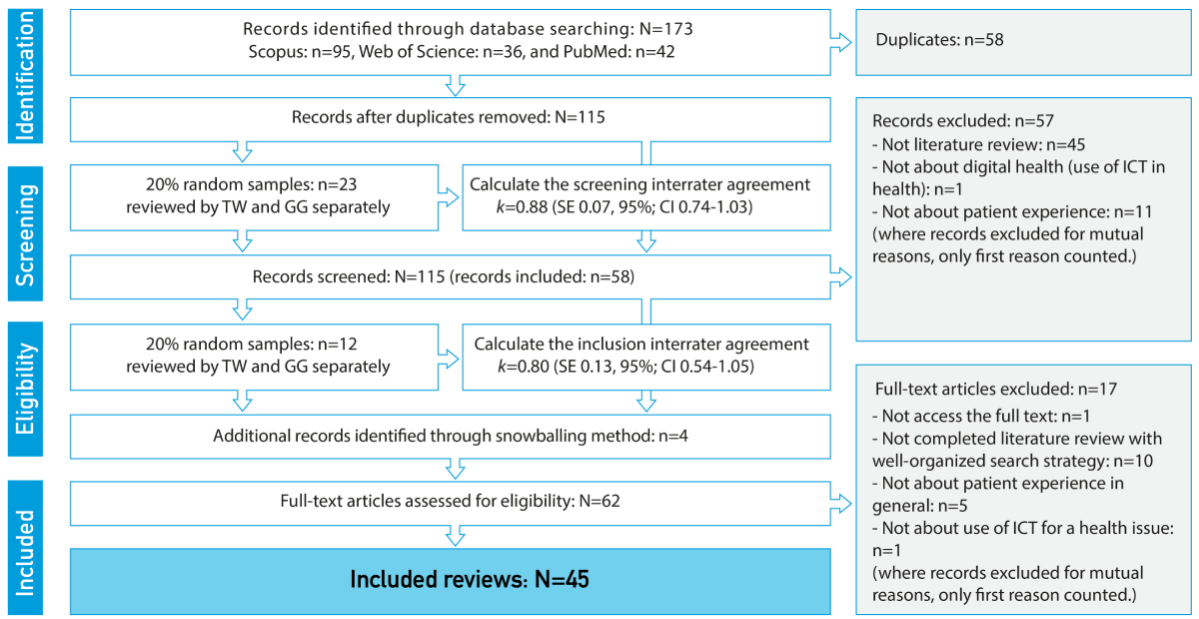
Study flow diagram. ICT: information and communication technology.

Following the PRISMA (Preferred Reporting Items for Systematic Reviews and Meta-Analyses) guidelines [50], we conducted an umbrella systematic review [51] on literature reviews related to PEx and UX in DH. The term DH was first introduced in 2000 by Frank [52]. Therefore, Scopus, PubMed, and Web of Science databases were used for searching related articles that were published between January 1, 2000, and December 16, 2020. Furthermore, Google Scholar was used to search for additional studies that were identified during the review process through the snowballing method. The computer search resulted in 173 articles, of which 58 (33.5%) were duplicates. After removing the duplicates, the titles and abstracts of a small random sampling (23/115, 20%) were reviewed by 2 independent raters to assess the interrater reliability by using the Fleiss-Cohen coefficient, which resulted in *k*1=0.88 (SE 0.07; 95% CI 0.74-1.03). This was followed by a group discussion to reach an agreement on the selection criteria. Subsequently, the remaining titles and abstracts (92/115, 80%) were reviewed by TW individually. After screening the titles and abstracts, half of the articles (58/115, 50.4%) remained for the full-text review. Meanwhile, 4 additional articles were identified through snowballing and were included in the full-text screening. Another small random sample (12/62, 19%) was reviewed by the 2 raters to screen the full texts. After achieving interrater reliability, *k*2=0.80 (SE 0.13; 95% CI 0.54-1.05) and reaching a consensus on the inclusion criteria through another group discussion, TW reviewed the full texts of the remaining papers (50/62, 80%). Google Sheets was used for performing the screening process and assessments. Finally, as shown in Figure 1 [3], a total of 45 articles were included for data extraction. A detailed search strategy, selection criteria, and screening process can be found in our previously published study [3]. Multimedia Appendix 1 [53-97] presents the included and excluded articles.

#### Data Extraction and Thematic Analysis

We used ATLAS.ti (Scientific Software Development GmbH; version 9.0.7) for data extraction. Data were extracted for the three predefined objectives: (1) evaluation timing considerations, (2) evaluation indicators, and (3) evaluation approaches of the digital PEx. In addition, we collected data related to evaluation objectives among the included studies. Data analysis followed the 6-phase thematic analysis method proposed by Braun and Clarke [98,99]: familiarization, coding, generating themes, reviewing themes, defining and naming themes, and writing up. First, we became familiar with the 45 articles included in the study. Second, after a thorough review, TW started iteratively coding the data related to the predefined objectives based on existing frameworks, including the Performance of Routine Information System Management framework [100], monitoring and evaluation guide [29], measures of PEx in hospitals [37], and an overview of research methodology [101]. This resulted in 25 initial codes. After no additional new codes were identified, TW proposed a coding scheme to summarize the recurring points throughout the data. Then, GG, RG, and MM reviewed and discussed the coding scheme until they reached an agreement. Third, TW followed the coding scheme to code the data more precisely and completely and searched for themes among the generated codes. Fourth, TW, GG, RG, and MM reviewed and discussed these codes and themes to address any uncertainties. Fifth, the definitions and names of the generated themes were adjusted through team discussions. Finally, the analytical themes related to the evaluation timing, indicators, and approaches were produced and reported. Both deductive and inductive approaches [99] were used to identify and generate themes. Four researchers were involved in the review process.

We first highlighted the evaluation timing considerations in terms of *intervention maturity stages*, the *timing of evaluation,* and the *timing of data* *collection,* which were adopted from the description of the WHO and European Union (Table 1) [36,37].

We then determined the evaluation indicators and classified them into 3 categories (Table 2). *Intervention outputs* are the direct products or deliverables of process activities and refer to the different stages of evaluation that correspond to the various stages of maturity of the DHI. *Patient outcomes* describe the intermediate changes in patients, including patients’ emotions, perceptions, capabilities, behaviors, and health conditions as determined by DHIs in terms of influencing factors and interaction processes. *Health care system impact* is the medium- to long-term, large-scale financial (intended and unintended) effects produced by a DHI.

Finally, we concluded evaluation approaches in terms of study designs, data collection methods and instruments, and data analysis approaches (Table 3). According to the WHO [29], study designs are intended to assist in decision-making on evidence generation and clarify the scope of evaluation activities. Data collection and analysis are designed through an iterative process that involves strategies for collecting and analyzing data and a series of specifically designed tools [36].

**Table 1:** Initial codes of evaluation timing considerations of the digital patient experience.

Categories and initial codes		Description
**Intervention maturity stages [29,36,37]**		
	Efficacy	Assess whether the DHI^a^ achieves the intended results in research or controlled setting
	Effectiveness	Assess whether the DHI achieves the intended results in nonresearch or uncontrolled setting
	Implementation	Assess the uptake, institutionalization, and sustainability of evidence-based DHIs in a given context, including policies and practices
**Timing of the evaluation [36]**		
	Before intervention	A baseline test is performed before individuals adopt or implement the intervention. It assesses individuals’ initial status and their anticipated perception of the intervention
	During intervention	An evaluation performed during intervention’s use aims to monitor individuals’ real-time feedback and reactions
	After intervention	An evaluation that is performed right after or a long time after the completion of the interventions by individuals. It assesses individuals’ changes regarding using the intervention
**Timing of data collection [35,37]**		
	Immediate evaluation	Aims to collect real-time data on patients’ experiences during or immediately after their treatment
	Delayed evaluation	Aims to obtain more substantial responses after the intervention’s completion over a long period
	Momentary evaluation	Aims to collect transient information from individuals at a specific moment
	Continuous evaluation	Aims to gather feedback from individuals at different points along the care pathway

^a^DHI: digital health intervention.

**Table 2:** Initial codes of evaluation indicators of the digital patient experience.

Categories and initial codes		Description
**Intervention outputs [29,38-40,102]**		
	Functionality	Assess whether the DHI^a^ works as intended. It refers to the ability of the DH^b^ system to support the desired intervention.
	Usability	Assess whether the DHI is used as intended. It refers to the degree to which the intervention is understandable and easy to use.
	Quality of care	Assess whether the DHI delivers effective, safe, people-centered, timely, accessible, equitable, integrated, and efficient care services. It refers to the degree to which health services for individuals and populations increase the likelihood of desired health outcomes.
**Patient outcomes [11,41-45]**		
	Emotional outcomes	Assess whether patients’ feelings and well-being change positively or negatively because of the use or anticipated use of DHIs. It refers to what the patients feels.
	Perceptual outcomes	Assess whether the informed state of mind that patients achieve as intended before, during, or after using the DHIs. It refers to what the patient thinks and believes.
	Capability outcomes	Assess whether patients’ health literacy, communication skills, or computer confidence in managing diseases, communicating with health care providers, or operating digital devices increased as expected. It refers what the patient knows and acquires.
	Behavior outcomes	Assess whether patients engage in activities to cope with the disease and treatments through DHIs. It refers to what the patient acts and does.
	Clinical outcomes	Assess whether patients’ health improvements meet the intentions of the DHIs. It refers to what medical condition the patient is in and aims to maintain.
**Health care system impact [29]**		
	Economic outcomes	Assess whether the DHIs are cost-effective, whether the organization and DH users can afford the DHI system, and whether there is a probable return on investment. It refers to the use of health care resources.

^a^DHI: digital health intervention.

^b^DH: digital health.

**Table 3:** Initial codes of evaluation approaches of the digital patient experience.

Categories and initial codes			Description
**Study designs [29]**			
	Descriptive study	Aims to define the “who, what, when, and where” of the observed phenomena and include qualitative research concerning both individuals and populations.	
	Analytical study	Aims to quantify the relationship between the intervention and the outcomes of interest, usually with the specific aim of demonstrating a causative link between the 2, including experimental and observational studies.	
**Data collection methods and instruments [103]**			
	Qualitative methods	Qualitative research is expressed in words. It is used to understand concepts, thoughts, or experiences. Common qualitative methods include interviews with open-ended questions, observations described in words, and literature reviews that explore concepts and theories.	
	Quantitative methods	Quantitative research is expressed in numbers and graphs. It is used to test or confirm theories and assumptions. Common quantitative methods include experiments, observations recorded as numbers, and surveys with closed-ended questions.	
	Qualitative analysis	Qualitative data consist of text, images, or videos instead of numbers. Content analysis, thematic analysis, and discourse analysis are the common approaches used to analyze these types of data.	
	Quantitative analysis	Quantitative data are based on numbers. Simple math or more advanced statistical analysis is used to discover commonalities or patterns in the data.	

### Phase 2: The Updated Scoping Search

The decision to undertake an update of a review requires several considerations. Review authors should consider whether an update for a review is necessary and when it will be more appropriate [104]. In light of the “decision framework to assess systematic reviews for updating, with standard terms to report such decisions” [105], we consider that research on PEx in DH remains important and evolves rapidly. In case we missed some newly published articles that would bring significant changes to our initial findings, we conducted a rapid scoping search for articles published after our last search. We reran the search strategy as specified before with the addition of date (from December 16, 2020, to August 18, 2023) limits set to the period following the most recent search. After removing duplicates (73/367, 19.8%), we collected 294 articles in total. Following the same screening process and selection criteria, we finally identified 102 new eligible articles. The excluded articles were either not a literature review with systematic search (74/294, 25.2%), not about DH (87/294, 29.6%), not about PEx (26/294, 8.8%), our own parallel publications (2/294, 0.7%), or not accessible in full text (3/294, 1%). The eligible and ineligible articles in this phase are available in Multimedia Appendix 2. We found that the outcomes in the new studies were almost consistent with the existing data. For example, these articles either aimed to investigate what factors influence the feasibility, efficacy, effectiveness, design, and implementation of DH; examine how patients expect, perceive, and experience the DHIs; or intend to compare the DHIs with conventional face-to-face health care services. The research objectives of these new eligible articles are available in Multimedia Appendix 3. We considered that their findings were unlikely to meaningfully impact our findings on when to measure, what to measure, and how to measure digital PEx. As suggested by Cumpston and Chandler [104], review authors should decide whether and when to update the review based on their expertise and individual assessment of the subject matter. We decided to use these new articles as supplementary materials (Multimedia Appendices 2 and 3) but did not integrate them into the synthesis of this review.

## Results

### General Findings

This paper is a part of a larger study, and we have presented results related to study characteristics in a previous publication [3]. Multimedia Appendix 4 [53-97] provides detailed information regarding the characteristics of the included reviews, including research questions or aims, review types, analysis methods, number of included studies, target populations, health issues, and DHIs reported in each review. In this study, to achieve our research objectives, we identified reviews that reported different intervention maturity stages, timing of the evaluation, and timing of data collection. In addition, we identified a set of evaluation indicators of digital PEx and classified them into 3 predefined categories (ie, intervention outputs, patient outcomes, and health care system impact), which in turn included 9 themes and 22 subthemes. Furthermore, we highlighted evaluation approaches in terms of evaluation theories, study designs, data collection methods and instruments, and data analysis methods. We found that it was valuable to compare the evaluation objectives of the included studies. Therefore, we captured 5 typical evaluation objectives and the stakeholders involved, which clarified why and for whom DH evaluators carried out the evaluation tasks. The detailed findings are presented in the Evaluation Objectives section.

### Evaluation Objectives

Our review findings highlighted 5 typical evaluation objectives.

The first objective was to *broaden the general understanding of the digital PEx and guide evaluation research and practice* (11/45, 24%) [53-63]. For instance, 1 review [61] aimed to identify implications for future evaluation research and practice on mental health smartphone interventions by investigating UX evaluation approaches.

The second was to *improve the design, development, and implementation of the DHI in terms of a better digital PEx* (15/45, 33%) [53-62,64-68]. As demonstrated in an included review [58], the evaluation of DHIs is critical to assess progress, identify problems, and facilitate changes to improve health service delivery and achieve the desired outcomes.

The third was to *achieve evidence-based clinical use and increase DHIs’ adoption and uptake* (14/45, 31%) [53,55,56,58-62,64,65,67,69-71].

The fourth was to *drive ongoing investment* (3/45, 7%) [53,70,71]; without compelling economic supporting evidence, the proliferation of DHIs will not occur. Therefore, ensuring the sustained clinical use, successful implementation, and adoption of and continued investment in DHIs require more evaluative information. This helps ensure that resources are not wasted on ineffective interventions [53].

The fifth was to *inform health policy practice* (3/45, 7%) [55,56,61]. As the 2 included articles stated [55,56], ongoing evaluation and monitoring of DHIs is critical to inform health policy and practice. In addition, in terms of the varied evaluation objectives, the evaluation activities serve different stakeholder groups, including program investigators, evaluators, and researchers; designers, developers, and implementers; end users, patients, and health care providers (HCPs); clients and investors; and governments and policymakers.

### Evaluation Timing Considerations

Among the included studies, evaluations were carried out at various stages of the intervention to fulfill the 5 evaluation objectives. Our findings showed that most reviews reported feasibility, efficacy, and pilot studies (32/45, 71%) [53,54,56,58-64,67,69-89] and then investigated effectiveness (20/45, 44%) [53,58,60,61,63,65,69,70,72,75,79,80,82-84,86,​^90^-^93^] and implementation studies (20/45, 44%) [54,56,58,61,62,64,68,70,73,75,78,81-83,85,87,88,90,91,94]. Notably, some reviews included >1 type of study. Our findings show that the timing of evaluation can be directly at pre- or postintervention [53,56,59,60,62-65,69-72,77,78,81,82,84,85,​^87^,^88^,^90^,^91^], at the baseline point or after a short- or long-term follow-up intervention [58,59,62,65,67,70-72,76,77,79,81,​^82^,^84^,^85^,^88^,^91^,^95^,^96^], during intervention use [76,85], continued monitoring [56,75], and even at dropout [53]. One study [84] suggested providing a period of technical training and conducting a baseline test to reduce the evaluation bias caused by individual technology familiarity and novelty. As demonstrated by another study [53], pre- and postintervention assessments using clinical trials can measure intervention effectiveness (eg, patients’ blood glucose levels). In terms of the timing of data collection, 1 included study [53] suggested that evaluations directly after the intervention are appropriate so that the users retain fresh memories of the experience. To sustain intervention outcomes over a longer period, longitudinal evaluations and long-term follow-up evaluations were recommended in 2 studies [55,84].

### Evaluation Indicators

#### Overview

Evaluation indicators relate to the goal to which the research project or commercial program intends to contribute. Indicators are defined as “a quantitative or qualitative factor or variable that provides a simple and reliable means to measure achievement, to reflect the changes connected to an intervention, or to help assess the performance of a development actor” [106]. On the basis of our initial codes, we grouped the evaluation indicators into 3 main categories: intervention outputs, patient outcomes, and health care system impact. Each category contains several themes and subthemes (Tables 4-6) and is discussed in detail in the below 3 sections: Intervention Outputs, Patient Outcomes, and Health Care System Impact.

**Table 4:** Themes, subthemes, and evaluation indicators of the intervention outputs of the digital patient experience.

Themes and subthemes		Studies (n=45), n (%)	Evaluation indicators	References
**Functionality (n=36, 80%)**				
	Intended values	21 (47)	Ability to either change or maintain the user’s health state in a beneficial way: support self-management, shared decision-making, trigger actions, and track and respond to changes Ability to collect clinical metrics: the number of monitored variables and the frequency, accuracy, concordance, timeliness, and visibility of monitoring	[53,54,56,61-63,65-69,74,​^79^,^81^-^83^,^88^,^91^,^93^,^96^,^97^]
	Content and information	20 (44)	Quality of the content: evidence based, tailored, relevance, practicality, consistency, and clarity Amount of the information: comprehensible, completeness, glanceability (understandability), and conciseness Language of the information: simple nontechnical language; actionable message; and a nonauthoritarian, friendly, and nonjudgmental tone of voice	[54-56,59,61,64,65,67,68,​^71^,^76^,^80^,^81^,^83^,^84^,^86^,^89^,​^90^,^93^,^95^]
	Intervention features	20 (44)	Appropriate features that meet the intended values: activity planning, activity scheduling, activity tracking, diary, alerts, journal, feedback, and reminders Degree of setup, maintenance, and training: ready to use, initial training, and ongoing education Channel or mode of delivery: phone calls, social media, mobile apps, web, video, devices, and wearable kit	[53-56,60-65,67-69,76,​^80^,^81^,^83^,^86^,^89^,^94^]
	Theory-based interventions	11 (24)	Presence or absence of an underlying theoretical basis: behavior change theory, social presence, and a quality certification	[54,59,68,71,78,83,86,​^88^-^90^,^92^]
**Usability (n=26, 58%)**				
	Technology quality attributes	24 (53)	Technology operability: the ease of use, learnability, memorability, readability, efficiency, system errors, product, or service Technology standards and specifications: interoperability, integration, scalability, ergonomics, connectivity, adaptability, flexibility, accuracy, and reliability	[53-57,59-62,64,65,67,​^68^,^71^,^73^,^75^,^76^,^82^,^86^,^88^,​^91^,^93^,^95^,^96^]
	Interaction design	17 (38)	Use of human-centered design methodologies during the development process: co-design, user-centered design, and inclusive design Design quality of system architecture, layout, and interface: intuitive, interactive, personalized, and esthetic	[53-57,59-62,67,68,71,73,​^83^,^86^,^88^,^94^]
**Care quality (n=30, 67%)**				
	Accessible care	27 (60)	Accessibility of care services: data, information, and HCPs^a^ Involvement of related stakeholders: family, friends, and peer-to-peer communication Accessibility to high-quality care: timely, integrated, continuous, improved (more predictable daily life), convenient (fits into daily routines), and personalized care	[53-56,59,62-64,66-69,​^71^,^75^-^81^,^84^,86>,^88^,^91^,^92^,​^95^,^97^]
	Safe and credible care	14 (31)	Credibility and accountability of care: the owners’ credibility and third-party verification Security of care: the number of medical errors Privacy of care: the presence of general privacy notifications, the documentation of individual access to user private data, and regulation compliance	[53-56,67,68,71-73,79,80,​^92^-^94^]

^a^HCP: health care provider.

**Table 5:** Themes, subthemes, and evaluation indicators of patient outcomes of the digital patient experience.

Themes and subthemes		Studies (n=45), n (%)	Evaluation indicators	References
**Emotional outcomes (n=32, 71%)**				
	Positive emotions	31 (69)	Patient satisfaction A sense of reassurance Well-being A sense of security Peace of mind A sense of belonging	[53,55,57,61-67,69,71,72,74-78,​^81^,^82^,^84^,^85^,^88^-^92^,^94^-^97^]
	Negative emotions	16 (36)	Concerns Fears A sense of uncertainties Dissatisfaction A sense of frustration A sense of insecurity Worries	[55,61,62,64,66,67,71,76,78,85,​^86^,^88^,^91^,^92^,^95^,^97^]
**Perceptual outcomes (n=32, 71%)**				
	Empowerment	23 (51)	Perceived values Quality of life Confidence Self-efficacy Comfort	[53,55,56,61,62,65-67,69,71,74-78,​^84^,^85^,^88^-^92^,^95^]
	Acceptability	19 (42)	Degree to which technology, treatment, and care services are accepted: willingness to use, intention to use, intention to continue using, and likelihood to recommend	[53,56,59-62,65,67,69,72,74-76,​^78^,^80^,^82^,^86^,^91^,^95^]
	Connectedness	16 (36)	Relationships between patient and provider: closeness, detachment, trust, or doubts	[53,61,64,66-69,71,72,75-78,88,​^92^,^97^]
	Attitudes	14 (31)	Initial beliefs, preferences, and expectations Impression of the excellence of the DHIs^a^ Interpretation of the DHIs Motivation to change behavior	[53,55,64,65,67,69,72,74-78,80,88]
	Burden	12 (27)	Perceived burden and restriction Discomfort Unconfident	[55,66,67,72,76-78,85,89,92,95,97]
**Capability outcomes (n=19, 42%)**				
	Autonomy and knowledge-gaining	19 (42)	Participants’ level of informed state of mind after using the DHIs: clinical awareness Patients’ level of health knowledge: health literacy, skills, and understanding Patients’ ability to make clinical decisions: problem-solving and shared decision-making	[53,56,62,64,65,69,71,72,75-79,​^84^,^88^,^90^,^92^,^95^,^97^]
**Behavioral outcomes (n=26, 58%)**				
	Adherence	19 (42)	Initial, sustained use of certain features Download and deletion rates Completion rates Dropout rates Speed of task completion	[55,61-63,65,67,69,74-76,78,​^79^,^82^,^84^,^88^-^91^,^95^]
	Self-management behaviors	17 (38)	Number of individuals exercising regularly or using dietary behaviors compared with the total number of participants Engagement of treatment, self-care, and help-seeking behavior	[53,59,61,62,65,67,69,74,75,​^78^,^81^,^84^,^85^,^88^-^90^,^92^]
	Patient-provider communication	11 (24)	Number and frequency of patient-provider contacts Engagement of patient-provider communication Quality of patient-provider communication (eg, percentage of patients reporting that HCPs^b^ communicated well)	[59,63,64,69,71,75,77,79,81,88,92]
**Clinical outcomes (n=23, 51%)**				
	Health conditions	23 (51)	Level of pain and symptoms control Status of physical health Level of health or treatment-related anxiety, depression, and stress Mortality rates Morbidity rates Adverse effects	[59-65,67,69,71,74-76,78,79,81,​^82^,^88^-^91^,^95^,^96^]

^a^DHI: digital health intervention.

^b^HCP: health care provider.

**Table 6:** Themes, subthemes, and evaluation indicators of health care system impact of the digital patient experience.

Themes and subthemes		Studies (n=45), n (%)	Evaluation indicators	References
**Economic outcomes (n=16, 36%)**				
	Cost-effectiveness	14 (31)	Out-of-pocket expenses for patients: care costs and travel costs Time efficiency of using the DHIs^a^: waiting time, travel time, and consultation time Reduction in overuse of services: printed materials	[56,63,64,66,72,76,​^77^,^79^,^81^,^88^,^90^,^91^,^96^,^97^]
	Health care service use	8 (18)	Duration of consultations Number of hospitals, primary care, and emergency department visits Hospital admissions Hospitalization Proportion of referrals	[62,64,76,81,88,90-92]

^a^DHI: digital health intervention.

#### Intervention Outputs

Intervention outputs are partially determined by the intervention inputs and processes (ie, influencing factors and design considerations, such as personalized design) [3]. We identified 3 themes and 8 subthemes within this category (Table 2). The first theme, *functionality,* refers to the assessment of whether the DHIs work as intended. The subthemes included (1) the consistency of intended value (eg, the ability of the DHIs to collect the amount of accurate clinical metrics in real time [56,62,74,88]), (2) the quality of content and information (eg, tailored content [56,64,76,81,83,86,89,90]), (3) the appropriateness of intervention features (eg, the degree of system setup [54,69]), and (4) the use of intervention theories (eg, the presence of an underlying theoretical basis [54,59,68,78,83,86,88,90,92]). The second theme, *usability,* refers to whether the DH system is used as intended [29]. Both technology quality attributes (eg, ease of use [53-56,59,61,62,67,68,71,76,86,95]) and interaction design (eg, intuitive interface design [67,68,94]) can be used for usability evaluations. The third theme, *care quality,* refers to effective, safe, people-centered, timely, accessible, equitable, integrated, and efficient care services [102]. For example, the assessment of convenient care accessibility (eg, care that fits into daily routines [53,59,62,76,77,81,86,88] and the credibility of DHIs’ owners [53,54]).

#### Patient Outcomes

Studies used a variety of quantitative and qualitative factors and variables to measure and describe patient outcomes (Table 3), referring to 5 themes (emotional outcomes, perceptual outcomes, capability outcomes, behavioral outcomes, and clinical outcomes) and 12 subthemes. *Emotional outcomes* relate to patients’ positive or negative feelings that result from the use or anticipated use of DHIs. For example, a high level of patient satisfaction [53,55,57,61-67,69,72,74-76,82,84,89-91,94-96] is a typical positive feeling. Increased concerns about data privacy and security [55,64,67,71,76,86,95,97] is a frequently mentioned negative feeling. *Perceptual outcomes* are the informed states of mind or nonemotional feelings the patients achieve before, during, or after using the DHIs [69], including patients’ initial attitudes toward the DHIs (eg, internal motivation [53,64,69,75,77,78,88]); patient-to-provider relationships, for example, those that are enhanced by perceived improved accessibility to HCPs [53,67,69,71,75,76,78,88,92] versus those that are interfered with by perceived loss of face-to-face contacts [61,64,66,71,76,77,97]; perceived empowerment (eg, increased confidence in managing their health conditions [56,69,75,77,78,90]) and burden (eg, increased perception of restriction [55,76-78,85,92,95,97]); and overall acceptance of the DHIs (eg, willingness to use [61,62,67,72]). *Capability outcomes* refer to the improvement in patients’ self-management autonomy, health knowledge, and clinical awareness. DHIs may be effective at improving their independency, self-management autonomy, problem-solving, and decision-making skills [53,62,64,65,69,71,75-79,84,88,92,95]; gaining health literacy, knowledge, or understanding of their health conditions or care plans [53,56,72,75,79,88,90,92,97]; and raising their clinical awareness to be more certain of when it was necessary to seek medical attention [69,71,72,78,92]. *Behavioral outcomes* include activities that the patients adopt owing to DHIs [69], including adherence to the intervention (eg, dropout rates [61,65,69,74,76,82,84]), self-management behaviors (eg, physical and diet activities [65,67,74,78,84,88,89]), and patient-to-provider communication (eg, increased interactions between patients and HCPs [59,63,64,69,71,75,77,79,81,88,92]). *Clinical outcomes* are related to individual health conditions and the main intentions of the DHIs. For example, a reduction in anxiety, depression, and stress [59,61-65,69,71,75,76,78,81,82,89,95] and increased symptom control [67,69,71,75,78,88-90,96] can help to measure the individual health conditions.

#### Health Care System Impact

Health care system impact contains 1 theme and 2 subthemes. *Economic outcomes* refer to the cost-effectiveness and health care services use. In terms of cost-effectiveness, for example, studies report less out-of-pocket expenses for patients because of reduced care and travel costs [56,63,64,79,81,88,90,91,97] and greater time efficiency owing to shorter waiting, travel, and consultation time [66,72,76,77,81,91,96]. Furthermore, indicators related to health care service use, such as the reduced number of hospital [62,64,76,90,91] and emergency department visits [90,91], can be used to assess savings regarding health care services.

### Evaluation Approaches

#### Overview of the Approaches

In addition to evaluation timing considerations and indicators, strategies and specifically designed tools for collecting and analyzing data are required to set up the evaluation plan. Various evaluation approaches were identified based on our initial codes; these are depicted in 3 aspects (Tables 7-9): study designs, data collection methods and instruments, and data analysis approaches. Furthermore, we collected data related to evaluation theories that were used to guide the study designs, data collection, and analysis.

**Table 7:** Study designs for evaluating the digital patient experience.

Study designs		Studies, n (%)	References
**Mode of inquiry (n=36, 80%)**			
	Qualitative research Phenomenology Ethnography	35 (78)	[53,55,56,58,59,61,62,64-72,74-83,85,86,88,90-92,95-97]
	Quantitative research	21 (47)	[53,55,58,61,62,64,66-68,70,71,74,76,83,85,86,88,90,91,95,96]
	Mixed methods research (and multiple methods research)	17 (38)	[53,55,56,61-63,66,68,71,76,78,80,85,86,88,91,95]
**Nature of the investigation (n=33, 73%)**			
	Experimental research Randomized controlled trials Nonrandomized trials	25 (56)	[53,58-60,62-64,70-72,75,76,78-85,88-91,95,96]
	Observational research	9 (20)	[60,72,76,80,84-86,88,91]
	Descriptive research Case reports Case series Cross-sectional	7 (16)	[55,56,68,71,72,74,91]
	Analytical research Case control Cohort	6 (13)	[55,60,71,88,91,94]
**Number of contacts (n=21, 47%)**			
	Cross-sectional	8 (18)	[55,56,68,72,74,91]
	Longitudinal	6 (13)	[55,62,71,93,95,97]
	Before and after	4 (9)	[53,59,60,62-65,71,72,81,82,87,90]
**Reference period (n=10, 22%)**			
	Prospective	8 (18)	[60,62,71,72,81,89,91,94]
	Retrospective	4 (9)	[56,60,91,95]
**Research through design (n=4, 9%)**			
	User research	3 (7)	[55,60,87]
	Participatory design or contextual design	1 (2)	[69]
	Design sessions	1 (2)	[55]

**Table 8:** Data collection methods of evaluating the digital patient experience.

Data collection methods	Studies, n (%)	References
Questionnaires	33 (73)	[53,55,56,58,59,61,62,64-69,71,72,75,76,79-87,89-93,95]
Surveys	32 (71)	[53,55,58,59,61-69,71-74,76,77,79-83,86,87,89-91,93-95]
Interviews	31 (69)	[53,55,56,59-62,65-69,71-73,75-78,80-87,91,92,95,97]
Focus groups	19 (42)	[55,56,58-60,63,66-68,76,78,80,81,83,85-87,95,97]
Observations	17 (38)	[55,60,66,67,69,71,72,76,78,80,84-88,91,97]
Log data	13 (29)	[55,61,69,71-74,81,83,84,90,95,97]
Open-ended questions	10 (22)	[53,56,59,62,65,67,75,77,80,86]
Likert scales	10 (22)	[53,58,65,67,71,82,84,89,91,93]
Usability testing	8 (18)	[53,57,60,64,67,81-83]
Diaries	6 (13)	[53,55,68,80,90,97]
Contextual inquiry	5 (11)	[53,56,69,80,87]
Needs assessment	5 (11)	[53,77,82,83,87]
Performance tests	5 (11)	[60,61,65,72,84]
Field notes	4 (9)	[56,69,85,97]
Workshops	4 (9)	[67,68,82,83]
Forms	3 (7)	[53,72,82]
Think-aloud method	3 (7)	[53,68,69]
Benchmark testing	2 (4)	[61,87]
Human impact assessment methodologies	1 (2)	[95]
Personas	1 (2)	[87]

**Table 9:** Data analysis approaches of evaluating the digital patient experience.

Data analysis approaches	Studies, n (%)	References
Statistical analysis	15 (33)	[59-61,65,70-72,74-76,82,84,90,91,96]
Thematic analysis	11 (24)	[56,61,69,76,77,80,85,88,92,95,97]
Content analysis	9 (20)	[53,56,63,76,77,80,86,92,97]
Grounded theory	7 (16)	[53,56,61,80,85,92,97]
Framework analysis	5 (11)	[56,80,85,92,97]
Heuristic analysis	4 (9)	[61,67,80,87]
Cost analysis	4 (9)	[63,70,88,91]
Task analysis	3 (7)	[61,83,87]
Text analysis	2 (4)	[66,92]
Document analysis	2 (4)	[71,80]
Failure analysis	2 (4)	[83,87]
Inductive analysis	2 (4)	[56,97]
Deductive analysis	1 (2)	[56]
Formal analysis	1 (2)	[73]
Decision analytic approach	1 (2)	[91]

#### Evaluation Theories

Our findings showed that in some cases, theories are used to guide the evaluation process. An included review [58] mapped various DHI evaluation frameworks and models into conceptual, results, and logical frameworks as well as theory of change. Among the included reviews, the National Quality Forum [63,79], UX model [93], American Psychiatric Association App Evaluation Model [61], Markov model [88], and Consolidated Framework for Implementation Research [56] were mentioned as evaluation frameworks or models for setting up, conducting, or analyzing the evaluation activities. In addition, theories from other fields such as frameworks or models related to *health care* (eg, diabetes theory [56,69], triple aims framework [91], and chronic disease management model [58]), *behaviors* (eg, social cognitive theory [59,82,93], behavior change theory [58,59,90]), *design* (eg, human factors principles [87], and inclusive design [57]), and *technology* (eg, the Unified Theory of Acceptance and Use of Technology [57,64] and Health Information Technology Usability Evaluation Model [67]) can be adopted to assess specific outputs, outcomes, or impact. For example, the behavior change theory can be used to guide the evaluation of patient behavioral outcomes [59].

#### Study Designs

The terminologies used to describe the study designs were mixed in terms of different classification bases. Following the work on research methodology by Kumar [101], we identified 4 standards for classifying study designs in DH: the perspectives of mode of inquiry, nature of the investigation, reference period, and number of contacts with the study population. From the perspectives of “mode of inquiry,” we found 3 types of study. The first used a qualitative study design, such as phenomenology or ethnography studies. The second were quantitative studies. The third type used mixed methods research and multiple methods research (ie, >1 qualitative or quantitative method, such as using both focus groups and interviews to collect data). In addition, based on *the nature of the investigation*, the collected primary studies among the included reviews were reported as observational studies versus experimental studies (RCTs and nonrandomized trials) and descriptive studies (case reports, case series, and cross-sectional) versus analytical studies (case-control or cohort studies). On the basis of the *number of contacts* with the study population, cross-sectional, before-and-after, and longitudinal studies were mentioned. Furthermore, in terms of the *reference period* (the time frame in which a study explores a phenomenon, situation, event, or problem), some studies included prospective designs, whereas others reported retrospective study designs. In addition, we note that others reported study designs from a design perspective, such as user studies, participatory design or contextual design, and design sessions.

#### Data Collection Methods and Instruments

Various data collection methods were used among the included reviews: questionnaires, surveys, interviews, focus groups, observations, log data, open-ended questions, Likert scales, usability testing, diaries, contextual inquiry, needs assessment, performance tests, field notes, workshops, forms, think-aloud method, benchmark testing, human impact assessment methodologies, and personas. Notably, these data collection techniques appeared as a mixed combination in some studies. In addition, we found various standard evaluation tools and performance tests used to collect the digital PEx–related data in 18 of the included papers [53,55,57,59,61,65,67,71,75,76,79,82,84,87,89,91,94,96], including the System Usability Scale [53,61,82], Patient Activation Measure [75,84], Patient Health Questionnaire-9 [75,89], and Beck Depression Inventory [75,89]. However, none of these tools are designed for evaluating the digital PEx; most are designed or modified to evaluate UX, PEx in general, or the usability of specific DHIs.

#### Data Analysis Approaches

Our findings showed that different types of data were used to evaluate digital PEx, such as self-reported data [74] and observable or monitored data [61]. To analyze the evaluative information, various data analysis methods were reported among the included reviews, including statistical analysis, thematic analysis, content analysis, grounded theory, framework analysis, heuristic analysis, cost analysis, task analysis, text analysis, document analysis, failure analysis, inductive analysis, deductive analysis, formal analysis, and decision analytic approach.

## Discussion

### Principal Findings

The goals of this umbrella review were to systematically review the evaluation timing considerations, indicators, and approaches of digital PEx. Furthermore, we identified 5 typical evaluation objectives and related audiences. The timing of a digital PEx evaluation should be a critical consideration when conducting an evaluation study; however, we found limited information about when to measure digital PEx. Moreover, the identified evaluation indicators are often heterogeneous and appear to be related to the different aspects of digital PEx. In terms of evaluation approaches, various theories were reported in the included papers. Furthermore, we noted that not only did the evaluation methods differ between the reviews but also the classification bases or perspectives used to describe these methods. Following our findings on when to measure, what to measure, and how to measure digital PEx, we generated a step-by-step evaluation guide and proposed 6 research directions for future studies.

### When to Measure

DHIs change throughout the product life cycle, so to provide better-quality results and evidence-based health practice, evaluations need to be incorporated into the intervention maturity stages [55,56,58,80]. Our findings showed that many studies were not performed in a real-world setting for a long period, and most studies were either feasibility or pilot studies; these results are directly in line with previous findings [56,65,73,82,85,89,97]. Pilot or feasibility studies can help improve new intervention development but only provide limited evidence for increasing sustained clinical use and large-scale practice [58,80]. Two studies [55,77] reported a lack of information on the long-term experience. Others have shown that some solutions may be less sustainable outside the trial context [80,85]. In addition, it is possible that participants were more adherent during the study period and decreased their use of the apps over time [74]. Therefore, some authors call for further research on digital PEx when incorporating the DHIs into existing health care services and processes [76]; there is a need to move DHIs from promise into policy and practice [56].

One study [72] reported significantly different evaluation results before and after the treatment. It is likely that patients’ initial emotional state or understanding of DHIs may affect their final PEx evaluation outcomes. Therefore, a baseline test on individual differences would be a valuable step to limit evaluation bias, as noted in a previous study [84]. We found that the data gathered could occur at a specific moment or at different time points along the care pathway to reflect a rapid or delayed digital PEx. Thus, posttreatment evaluations should account for the recall bias caused by the time delay between treatment and recollection of experience, as has been noted in previous studies [53,76]. In line with other studies [29,64], we believe that real-world testing and direct feedback from actual users will help improve the usability of DHIs and directly benefit new users.

### What to Measure

In comparison with intervention outputs and health care system impact, we discovered more evaluation indicators related to patient outcomes. We assume that this is owing to the consideration of the strength of the evidence and duration of the study. Patient outcomes enable the identification of patients’ actual experiences and reactions in uncontrolled settings, providing evidence for clinical use and further improvements. However, intervention outputs seem more suitable for exploring experts’ (eg, designers, health care professionals, and policymakers) or patients’ anticipated understandings of DHIs in the early stages of design and for addressing any potential system barriers. The health care system impact can be useful in predicting the sustainability of the DHIs on a large scale through a long-term study.

We used a set of themes and subthemes to describe each category. For instance, patient outcomes include emotional, perceptual, capability, behavioral, and clinical outcomes, as noted in 2 studies [60,75]: one study categorized the variables of patient engagement as behavioral, cognitive, and emotional outcomes, whereas the other study used biomarkers, perceptions, and behaviors to describe patient clinical outcomes with regard to DHIs. Furthermore, we noted that the evaluation outcome of one indicator is often unable to anticipate the outcome of another indicator. For instance, some patients reported high acceptance of a certain DHI, but they rarely used it [95]. Aligned with the arguments among the differences between patient satisfaction, PEx, PREMs, and PROMs [35,37,47,48], our findings indicate that digital PEx evaluations are not equivalent to the measurement of patient satisfaction, PEx, PREMs, or PROMs, but that these measures can be used to assess some of the digital PEx. We showed that the priorities of the evaluation indicators can differ between projects. In terms of what to measure first, as stated in a previous study [107], the goal of evaluations should be to focus on those processes that should be optimized by the digital catalyst. Furthermore, the evaluation indicators need to be continually updated as the DH landscape is rapidly evolving and the technology infrastructure is constantly shifting [54].

### How to Measure

As demonstrated in an included review [58], an evidence-based theoretical evaluation framework is helpful in informing the evaluation process. Across the included reviews, we found that not only specifically designed evaluation theories were used to guide the evaluation activities but also theories from other fields were adopted to assess the evaluative data. We identified various traditional approaches across the included reviews. In addition, our results showed that more than half of the included reviews reported RCTs in their studies. RCTs were recommended in 2 reviews [108,109] to evaluate DHIs for stronger evidence. However, a recent systematic review [110] noted that only a handful of clinical decision support systems have been tested in this way. Others argued that there is a tension between the amount of time needed for evidence generation with traditional approaches and the speed of digital product development and iterative upgrading [16,18], which requires more innovative methods for fast evidence generation [18].

We identified a wide range of evaluation methods and instruments, although most were modified based on the evaluations for traditional face-to-face treatment or usability testing in human-computer interactions. This is also in line with the findings from previous studies [53,61,72,79]. Semistructured interviews and questionnaires were the most common evaluation methods for collecting evaluative data among the included reviews, which is in line with previous studies [53,76]. Semistructured interviews are the key methods used to understand the details of UX [59,61,62,65,66], whereas questionnaires are often modified from existing assessments to assess large-scale interventions [53,61]. It is likely that more in-depth, observational data collection methods are necessary to better capture experience data [53,66]. The use of a descriptive approach might be appropriate for a smaller sample size, collecting qualitative data through surveys, focus groups, and interviews [76]. Standard functional questionnaires may be preferred when DHIs are compared with other interventions [53]. However, we found that detailed interview outlines or questionnaires were generally not published, as mentioned in another study [53]. Comprehensive information on user evaluation methods and results is often lacking [65]. The determination of evaluation approaches depends on the specific context. In alignment with 2 studies [4,40], we state that the choice of evaluation approaches heavily depends on evaluation objectives, timing, indicators, and evaluation requirements and resources. An included review [58] recommended using multiple research methods, such as combining qualitative, quantitative, co-design principles, and process measures, for evaluation designs.

Thanks to the use of digital technologies [54,75], patients’ illness experience and what they feel when participating in a health care intervention can be monitored. However, we found that these may blur the boundaries between interventions, monitoring, and evaluations. For example, the diary function can be used as an intervention feature (eg, a self-management diary to track symptoms and identify exacerbations [78]), as a monitoring tool (eg, diary entries [97] or adherence [90]), or as an evaluation method (eg, to capture user feedback [53]). Furthermore, a study indicated that with the advancement of technology, the ability of DHIs to collect “passive data” for assessing digital PEx may gain more attention and eventually eclipse the utility of DH-aided self-report [74]. Finally, we believe that involving multiple stakeholders is not only essential in the design process but is also a requirement for the evaluation process. Both end users and experts can contribute to the evaluation activities [53]. This aligns with a recent study that suggests that digital solution evaluation requires collective efforts from multiple parties, such as health authorities, HCPs, and manufacturers [18].

### Design Implications

Our analysis showed that the evaluation of a DHI follows the same evaluative process as that of traditional interventions, which supports a previous study [53]. To make the evaluation findings more comparable, more rigorous studies and standardized evaluations are suggested, including unified terminology [53,65,68], predefined measurable indicators [79,81], standardized methods [61,66], validated instruments [84,96], uniform time intervals [84], and adequate patient selection [81]. Intervention characteristics (eg, aims, expected outcomes, elements, length, frequency, and duration), study designs (eg, sample size, period, regulations, investigator, evaluators, recruitment, ethics, topic guides, or questions asked by the researchers), objectively measured patient health outcomes, and adverse events should be carefully considered when conducting and reporting an evaluation study [53,60,69,84].

**Figure 2:**
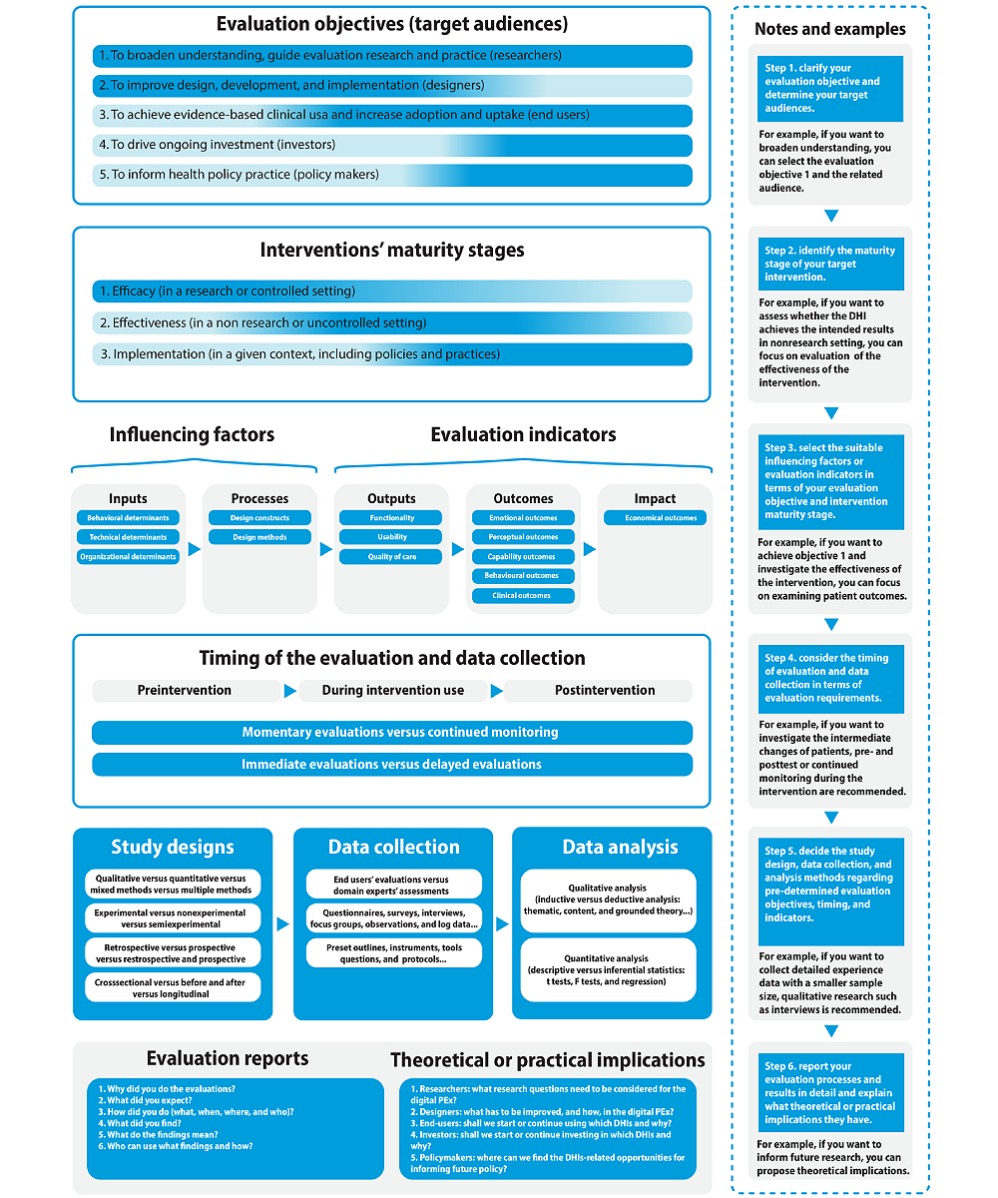
Digital patient experience evaluation guide.

Inspired by the challenges for the evaluation of DHIs [18]; shaped by the Performance of Routine Information System Management framework [100], the monitoring and evaluation DHIs guide [29], PEx measures [37], and our previous publications on influencing factors and design considerations of digital PEx [3,111]; and based on the findings of this study, we have developed a step-by-step evaluation guide for DH innovators, such as designers, developers, and evaluators (Figure 2): The first step is to clarify the evaluation objectives and determine the target audiences for the evaluation. We proposed 5 typical evaluation purposes and their related audiences. The selection of evaluation objectives can help determine the stages for evaluating the DHI. For example, we consider effectiveness and implementation studies more appropriate for achieving evidence-based clinical use and increasing adoption and uptake compared with efficacy studies. The second step is to determine the intervention contexts and foci in terms of the intervention maturity stages, including efficacy, effectiveness, and implementation. The determination of the evaluation stage is not only because of the evaluation objective but also because of the current condition of the DHI. The determination of the evaluation objectives and identification of the evaluation stage affect the consideration of influencing factors and evaluation indicators at the next step. For example, the evaluation of patient outcomes in an uncontrolled setting can provide evidence for clinical use and further improvement. The third step includes a set of influencing factors (ie, inputs and processes) and evaluation indicators (ie, outputs, outcomes, and impacts) that can be used for further formulating evaluation constructs. The former is more appropriate for formative evaluations, which often occur during the design and development process, whereas the latter is suitable for summative evaluations, which often occur during and after the implementation process. In the fourth step, we present 2 types of evaluations. On the basis of the frequency of evaluations, we can capture momentary experiences before, during, and following an intervention or monitor continuous feedback throughout the intervention. With regard to the time interval between the intervention and evaluation, assessments can reflect immediate experiences directly after the intervention or recalled experiences over an extended period. In the fifth step, we present various evaluation approaches that can be used to plan and carry out specific evaluation activities, such as study designs, data collection methods and instruments, and data analysis approaches. The consideration of study designs often affects the strength of the evidence and determines the data collection and analysis methods. Behavioral data may provide stronger evidence than opinion data. Qualitative methods, such as interviews, are more appropriate for collecting in-depth experience data for a smaller sample size in the early intervention development stages, and quantitative methods, such as questionnaires, are more suitable for investigating experience data at a large scale or comparing it with other interventions during or after the implementation stages. In the sixth step, we proposed 6 questions for the evaluation investigators to guide them in reporting the evaluation results and 5 questions to inspire them to generate theoretical or practical implications for responding to the related stakeholder groups. The answers to these 11 questions should reflect the evaluation processes and serve the evaluation objectives.

The guide can be used when setting up a digital PEx evaluation plan or guiding evaluation practice. Notably, the interrelationships between these 6 steps are not fixed; the entire evaluation plan is an iterative process; and the decisions made at the previous steps may influence the following steps, and vice versa. In addition, other considerations beyond this guide can also impact the evaluation process, such as human, time, and financial resources. Our guide presents an ideal way to conduct the evaluation of digital PEx; however, in the real world, the order of these steps may be changed or some steps may even be skipped depending on the specific project context. For instance, in certain assessment procedures, selecting an evaluation construct, such as usability, may come first, rather than taking evaluation objectives or target audiences into account. We developed this guide based on our literature analysis. It provides an overview of the most common evaluation timing considerations, indicators, and approaches used to collect digital PEx–related data. However, it may be incomplete and require updating in the future. For example, owing to the methodological limitations, we did not provide concrete recommendations on which evaluation approaches are superior for what types of DHIs. We believe that without providing a specific context and concrete project requirements, it is difficult to draw a conclusion.

### Limitations

This study had several limitations. First, we noted possible resource restrictions and the newness of the field, which may have led to missing articles. To overcome this, we searched 3 databases and used the snowballing method. In addition, we performed an updated literature search to check whether there were any meaningful new insights that would significantly change our conclusions. To our knowledge, although there were some newly published reviews in this area, we confirmed that our results were quite stable, and the newly identified studies were unlikely to significantly impact our results. Second, we could not perform a quality assessment because of the diversity in reviews and methodological limitations. As previous studies on investigating umbrella reviews have indicated, there are currently no official standards for determining the certainty of evidence when performing umbrella reviews [112,113]. In addition, among the included reviews, only 53% (24/45) of the studies assessed the risk of bias and used diverse quality assessment instruments. After a thorough attempt, we found that none of these instruments were suitable for assessing the various reviews included. These encompass systematic reviews, scoping reviews, comprehensive overviews, and general literature reviews, which incorporate various primary and secondary studies extending beyond RCTs and nonrandomized studies of interventions. This diversity makes it challenging to use a standardized method for assessing the quality of evidence across the extensive range of included reviews. However, we tried to reduce the risk of bias by only including reviews that were published in peer-reviewed journals. Third, reviewing secondary research may have led to the omission of crucial information and reporting bias. To minimize potential bias, we used the most common terms used across the included papers as themes and subthemes. Owing to the cross-disciplinary nature of the topic, there is a lack of consistency or clarity in the terminology used to describe the evaluation indicators and approaches. For instance, in one study [55], a user study was pitched at the same level as interviews or observations, whereas in another study [56], interviews and questionnaires are methods that form part of “user study” research. In addition, information related to the evaluation approaches was reported at different levels among the included studies. For example, one study provided information related to data collection methods, such as focus groups, design sessions, and questionnaires [55], whereas another study reported information related to study designs, including qualitative, quantitative, and mixed methods designs [71]. These inconsistencies complicated the comparison between different studies. To counter this, we analyzed the different classification bases behind these study designs. Finally, we could not draw firm conclusions regarding which evaluation approaches are better suited for which types of DHIs. Owing to the nature of this study being a review of reviews, details such as the characteristics of DHIs are not always adequately covered in each included review. In addition, the included reviews contained a large number of primary studies, which makes referring back to each primary study challenging. The lack of details about the characteristics of these primary studies limits the classification of DHIs in this study. Moreover, the included reviews represent a wide range of studies, making comparison across the included reviews challenging. In addition, we decided that this was out of the scope of this study. When planning this study, we deemed it more appropriate to initially offer an overview of diversities rather than begin with a best practice recommendation. Consequently, we aimed to map possible evaluation considerations and approaches for evaluating digital PEx, instead of discussing which approach is better. However, we encourage future research to address this issue.

### Future Research

Considering our research limitations, to further facilitate evaluations of digital PEx, we propose 6 future research directions. First, further research into how one indicator mediates another indicator’s impact on digital PEx is required. For example, is there a correlation between clinical outcomes and perceptual outcomes? To explore this, we performed an experimental study to investigate whether patients’ initial pain perception and technology acceptance (using virtual reality distraction) affected their experienced pain during wound care treatment. Our findings will be published in a future article. Second, the variables that influence the selection or prioritization of evaluation indicators and approaches should be further investigated. For example, it would be valuable to investigate whether some evaluation indicators and approaches are better suited for evaluating certain types of DHIs according to the strength of the evidence and the length of the evidence generation time. Third, agreement is needed on standardized measures to evaluate digital PEx, particularly innovative approaches for faster and high-quality evidence generation. In a follow-up interview study, we aim to summarize the often-used agile evaluation approaches based on designers’ experiences. Furthermore, in cases where an interview or questionnaire is used to collect evaluative information, we recommend reporting the detailed interview outlines or questionnaires together with the evaluation results. Fourth, research is needed on how the intervention maturity stages and timing of the evaluation of the evaluation affect the evaluation results. Fifth, future studies should not only investigate whether DHIs achieve the intended results in a research setting but also assess the long-term digital PEx regarding the uptake, institutionalization, and sustainability of evidence-based DHIs in a given context and a real-world setting, including policies and practices. Finally, research is required on how to analyze and respond to the evaluative data. We recommend that future evaluation research and practice provide theoretical and practical guidance on how to use the evaluative information.

### Conclusions

To effectively improve the digital PEx, knowing how to evaluate the digital PEx is as important as knowing what factors influence the digital PEx and how to design the digital PEx. Evaluating digital PEx requires clarifying the evaluation objectives, identifying stakeholder groups, considering reasonable evaluation timings, choosing relevant evaluation indicators, and selecting appropriate evaluation approaches. Following our previous publication on the influencing factors and design considerations of digital PEx [3], we first identified 5 typical evaluation objectives and related stakeholder groups. We then described potential evaluation timing considerations in terms of 4 intervention maturity stages and 3 evaluation timings. We collected knowledge on evaluation indicators of digital PEx and grouped them into 3 categories: intervention outputs, patient outcomes, and health care system impact. These were then classified into 9 themes (intervention functionality, usability, care quality, patient emotional outcomes, perceptual outcomes, capability outcomes, behavioral outcomes, clinical outcomes, and system financial outcomes) and 22 subthemes. Furthermore, we noted a set of common study designs, data collection methods and instruments, as well as data analysis methods, which can be used or adapted to evaluate digital PEx. On the basis of our findings, we developed an evaluation guide to help DHI researchers, designers, and developers further evaluate digital PEx. Finally, we recommend 6 directions for further research on digital PEx evaluation. Multimedia Appendix 5 (the PRISMA checklist) provides more detail on the structure of this review.

## Appendix

Multimedia Appendix 1 The first and second rounds of the review.

Multimedia Appendix 2 Results of a rapid scoping search.

Multimedia Appendix 3 The research aims or questions of the newly eligible reviews.

Multimedia Appendix 4 Study characteristics and digital health intervention characteristics of the included reviews.

Multimedia Appendix 5 PRISMA (Preferred Reporting Items for Systematic Reviews and Meta-Analyses) 2020 checklist.

## Abbreviations

DH: digital health
DHI: digital health intervention
HCP: health care provider
PEx: patient experience
PREM: patient-reported experience measure
PRISMA: Preferred Reporting Items for Systematic Reviews and Meta-Analyses
PROM: patient-reported outcome measure
RCT: randomized controlled trial
UX: user experience
WHO: World Health Organization
